# Plasma Membrane Integrates Biophysical and Biochemical Regulation to Trigger Immune Receptor Functions

**DOI:** 10.3389/fimmu.2021.613185

**Published:** 2021-02-19

**Authors:** Tongtong Zhang, Wei Hu, Wei Chen

**Affiliations:** ^1^Department of Hepatobiliary and Pancreatic Surgery, The Center for Integrated Oncology and Precision Medicine, Affiliated Hangzhou First People’s Hospital, Zhejiang University School of Medicine, Hangzhou, China; ^2^Department of Cell Biology and Department of Cardiology of the Second Affiliated Hospital, Zhejiang University School of Medicine, Hangzhou, China; ^3^Key Laboratory for Biomedical Engineering of Ministry of Education, State Key Laboratory for Modern Optical Instrumentation, College of Biomedical Engineering and Instrument Science, Collaborative Innovation Center for Diagnosis and Treatment of Infectious Diseases, Zhejiang University, Hangzhou, China

**Keywords:** immune receptor, plasma membrane, biophysical-chemical coupling, electrical potential, mechanical force

## Abstract

Plasma membrane provides a biophysical and biochemical platform for immune cells to trigger signaling cascades and immune responses against attacks from foreign pathogens or tumor cells. Mounting evidence suggests that the biophysical-chemical properties of this platform, including complex compositions of lipids and cholesterols, membrane tension, and electrical potential, could cooperatively regulate the immune receptor functions. However, the molecular mechanism is still unclear because of the tremendous compositional complexity and spatio-temporal dynamics of the plasma membrane. Here, we review the recent significant progress of dynamical regulation of plasma membrane on immune receptors, including T cell receptor, B cell receptor, Fc receptor, and other important immune receptors, to proceed mechano-chemical sensing and transmembrane signal transduction. We also discuss how biophysical-chemical cues couple together to dynamically tune the receptor’s structural conformation or orientation, distribution, and organization, thereby possibly impacting their *in-situ* ligand binding and related signal transduction. Moreover, we propose that electrical potential could potentially induce the biophysical-chemical coupling change, such as lipid distribution and membrane tension, to inevitably regulate immune receptor activation.

## Introduction

The plasma membrane (PM) of cells, mainly consisting of lipid, cholesterol, and protein, is a lipid bilayer structure. Its outer leaflet enriches phosphatidylcholine, sphingolipid, and cholesterol, and the inner leaflet mainly contains cholesterol and acidic phospholipids (e.g. phosphatidylserine, phosphatidylinositol, and phosphatidic acid) (1–3). The asymmetry mobility and dynamic organization of lipid and membrane proteins have been proposed in the Fluid-Mosaic model (4–7). In this model, the PM is a very dynamic structure, where lipid-protein, lipid-lipid, and protein-protein interactions occur at all times, and all of these interactions regulate membrane receptor’s ligand recognition and triggering (6, 8). Moreover, sphingolipid and cholesterol contribute to the formation of nanodomains or lipid rafts, which are highly dynamic in many receptor-activated cellular processes (9–12). It has been reported that the biophysical-chemical properties of the PM, including the asymmetry of lipid and protein distribution, the membrane curvature and mechanical tension, and the membrane electrical potential, could dynamically regulate diverse cellular processes (13–16).

For immune cells, the PM tunes their essential physiological processes. For example, the cholesterol accumulation could increase T cell differentiation and proliferation, whereas it also induces T cell exhaustion through T-cell receptor (TCR) signaling (17–20). Phosphatidylserine directly tunes T cell migration, adhesion, tissue infiltration, and rapid inflammatory response (21, 22). Moreover, PM’s mechanical tension, driven by the cytoskeleton and its associated molecular motors, dynamically shapes PM morphology and regulates T cell adhesion, migration, and activation cooperatively *via* many immune receptors (e.g. TCR and integrin) (23–25). Also, PM morphology (e.g. microvilli) facilitates the discrimination of peptide major histocompatibility complex (pMHC) for TCR (26–28). Membrane potential, another PM biophysical property, might also regulate T cell proliferation and cytotoxicity through TCR activation (29, 30).

Here, we review how PM couples with biophysical and biochemical factors to regulate the functions of immune cells (e.g. T cell, B cell, and natural killer cell) through the respective immune receptor activation, such as TCR, B-cell receptor (BCR) or Fc receptor (FcR), and further discuss and propose the potential molecular mechanism.

## Double-Edged Regulation of Cholesterol

It has been reported that many receptors (e.g. acetylcholine receptor and G protein-coupled receptor) contain the cholesterol recognition/interaction amino acid consensus (CARC, mainly containing Valine, Isoleucine, Alanine, Methionine, and Serine amino acids) motif in the transmembrane domain (TMD) which directly interacts with cholesterol (31, 32). This CARC motif might be conserved for many immune receptors. Based on previous findings, we propose a double-edged model of cholesterol regulation on receptor activation: 1) cholesterol directly binds TMD to keep immune receptors in an inactive (close conformation) state (33); 2) once the immune cell is stimulated, cholesterol indirectly mediates the clustering of immune receptors (34, 35), which might be in an activation (open conformation) state. Cholesterol might finely tune the activation threshold to avoid perturbations from non-specific noise signals. Once strong stimulation activates the conformational change of receptor TMD, cholesterol could facilitate immune receptors clustering to launch and amplify downstream signaling cascades. Therefore, whether and which residues of receptor TMD mediate direct interaction with cholesterol, and if so, how cholesterol keeps immune receptors in the close conformation or resting state, and how strong stimulation (e.g. ligand binding) could trigger the conformational change of immune receptors to form the cholesterol-mediated nano or micro clusters, need to be further investigated with atomic resolutions.

**Figure 1:**
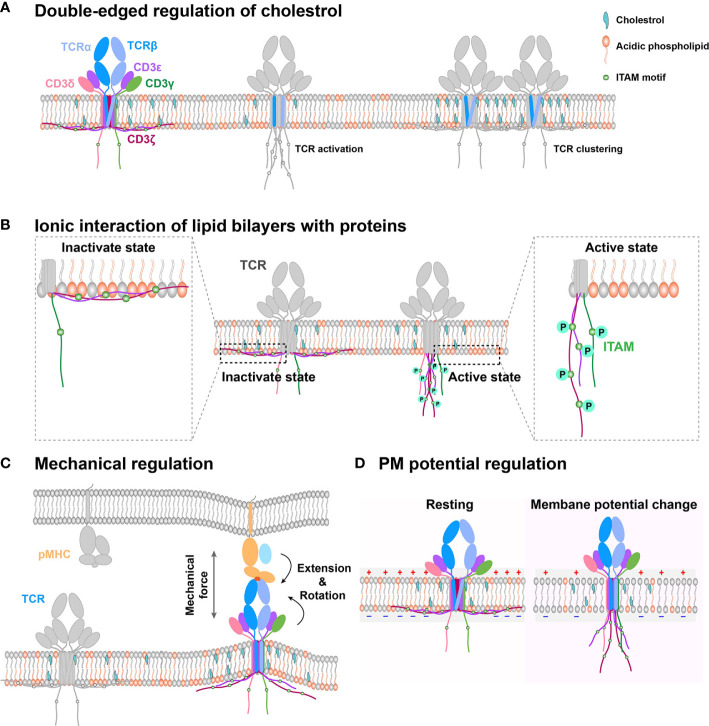
Schematic models of PM regulation on TCR complex signaling. **(A)** Double-edged regulation of cholesterol on TCR activation. Cholesterol could directly bind with the TMD of the TCR β chain to keep TCR in an inactive state in the resting T cell. Cholesterol disassociation from the TCR β chain can switch the TCR complex to the activation state. Meanwhile, cholesterol also indirectly mediates TCR clustering, following TCR initial activation. **(B)** The interaction between negatively charged lipid and basic motif regulates CD3 ITAM motif exposure. TCR cytoplasmic domains contain polybasic regions, which directly interact with the negatively charged lipid in the membrane inner leaflet to embed the ITAM motif in hydrophobic core of the PM in resting cell. The disruption of this interaction can expose the signaling motif to amplify downstream signaling. **(C)** PM provides a platform to sense outside cues for immune receptors. On this platform, mechanical force regulates TCR/pMHC recognition through conformation change. **(D)** Electrical potential might directly trigger TCR signaling. Since TCR TMD contains several charged residues, PM potential depolarization might induce TMD titling conformation to further allosterically regulate dissociation of CD3 tails from inner leaflet and activate intracellular downstream signaling.

As a major biochemical component of the PM, cholesterol can bifunctionally regulate TCR dynamics and functions (**Figure 1A**). On one hand, it associates with the TMD of the TCR β chain and keeps TCR in a resting and inactive conformation, preventing CD3 phosphorylation and recruitment of downstream signaling components, such as ZAP70 and ERK (33). On the other hand, it can also enhance TCR nanoclustering to promote T cell activation (36–38). Moreover, cholesterol can regulate TCR clustering and signaling through dynamic lipid rafts (35, 39, 40). For example, the increased cholesterol level in the PM by inhibiting cholesterol esterification of CD8^+^ T cell *in vivo* can promote TCR clustering, enhance immune synapse formation, and amplify the phosphorylation of CD3, ZAP70, and ERK to produce more cytokine, leading to T cell proliferation (41). Consistently, cholesterol sulfate can inhibit CD3 immunoreceptor tyrosine-based activation motif (ITAM) phosphorylation by replacing cholesterol to disrupt the formation of TCR nano-clustering (42). The depletion of cholesterol in T cells also drastically reduces *in-situ* TCR/pMHC binding affinities and association rates, potentially through regulating the conformation or orientation of TCR’s TMD and ectodomains to impair TCR antigen recognition (43). In brief, cholesterol possibly tunes TCR initial allosteric switch and subsequent clustering, respectively. The detailed regulation mechanism remains ambiguous, which requires further investigation with atomic resolution to reveal how exactly cholesterol dynamically associates with the TCR/CD3 complex. The cryo-EM structure of TCR/CD3 complex with membrane lipid and cholesterol will provide us more meaningful insights.

During the antigen recognition process of B cells, the micro-cluster formation of the BCR complex is crucial to strengthen BCR activation signaling (44, 45). Cholesterol has been reported to regulate the distribution of BCRs in PM microdomains, and low cholesterol level impairs BCRs aggregation further to affect Vav and Rac1 phosphorylation (46, 47). Moreover, cholesterol may affect the formation of protein islands, nanodomains, or microvilli on the PM, which provides a platform for BCRs to form their unique signaling complex with coreceptors (28, 48–50). Meanwhile, cholesterol could also induce BCR endocytosis on anergic cells to inhibit BCR signaling (51). Therefore, cholesterol also has double-edged regulation (amplifying or attenuating) on BCR signaling. However, the detailed molecular mechanism of these two regulatory effects and their switching is still unclear.

The lipid raft, mainly consisting with cholesterol, can directly tune activating receptor FcγRIIA signaling (e.g. phosphorylation of CbI and NTAL) without ligand binding (52). The cholesterol depletion can impair FcγRIIA association with CD55, GM1, and Lyn kinase, and the related phosphorylation signaling (53). Similar to FcγRIIA, FcγRIIIA activation could also be inhibited by cholesterol depletion to reduce ERK activation and prevent IFN-γ production (54). And the intracellular tyrosine phosphorylation of inhibitory receptor, FcγRIIIB, can also be significantly attenuated when the cholesterol level reduces (55, 56). Moreover, cholesterol can directly regulate the recognition of FcγRI to IgG (57). These detailed regulation molecular mechanisms still need to be further investigated.

## Signaling Motifs Protection by Negatively Charged Lipid/Basic Motif Interaction

The PM inner leaflet enriches negatively charged lipids (e.g. phosphatidylserine, phosphatidylinositol, etc.) that can interact with the polybasic regions of immune receptors to regulate their activation. Such interaction can embed the signaling motif of the immune receptors in the PM hydrophobic core. The positively charged Ca^2+^ ions flux, which is triggered by strong agonistic ligand stimulation, can disrupt this interaction to uncover the buried signaling site (58–61). The PM shields noise signal interference through their selective association with critical signaling motifs until strong stimulation is initiated. This mechanism of signaling shielding and amplification by regulating negatively charged lipid/basic motif interaction is potentially shared by many other immune receptors.

The inner leaflet of T cell PM mainly consists of negatively charged lipids, which associate with CD3ϵ/ζ cytoplasmic ITAM motif through electrostatic interactions (**Figure 1B**). These lipid and CD3 interactions protect CD3ϵ/ζ ITAMs from being recognized and phosphorylated by downstream kinase molecules, such as Lck (59, 60, 62, 63), thus keeping TCR/CD3 in the resting state and T cells in a quiescence state. As it has been extensively reviewed before (60), we here just briefly discuss it.

For the mIgG-BCR, PM’s inner side could block non-specific stimulation and keep mIgG-BCR in a resting state, providing the critical activation thresholds for mIgG-BCRs (58). As the cytoplasmic region of the mIgG (mIgG-tail) contains several basic residues, it could electrostatically bind with negatively charged acidic phospholipids of the PM’s inner leaflet to block the interference from noise signals and keep mIgG-BCR in a resting state in quiescent B cells (58). Ca^2+^ mobilization triggered by suitable antigenic stimulation on the mIgG-BCR complex could disrupt this protection, which further recruits pSyk, pBLNK, and pPI3K into the immunological synapse to induce a more potent Ca^2+^ mobilization response and B-cell hyperproliferation. Moreover, phosphatidylinositol (4,5)-bisphosphate (PIP2) triggers a signaling amplification loop to induce the initial formation of BCR micro-clusters upon B cell activation (64). PIP2 and phosphatidylinositol (3,4,5)-trisphosphate (PIP3) together tune the growth of BCR micro-clusters by recruiting Dock2 to remodel F-actin cytoskeleton (65). Remarkably, this electrostatical interaction mechanism might not be suitable for mIgG-associated Igα and Igβ as they contain polyacidic regions (58, 60).

## Mechanical Regulation and Its Coupling With Cholesterol and Negatively Charged Lipid

In general, the PM provides a platform for immune receptors to sense outside physical and biochemical cues. Unlike biomolecules in solution that can freely rotate and adopt many different possible orientations, immune receptors’ orientations are significantly restricted by the PM. They only can adopt limited protein topologies, which are essential for receptor-ligand binding and downstream signaling transduction. The membrane anchor pattern, extracellular length, and orientation of immune receptors all could influence the recognition of their ligands by affecting ligand accessibility and association kinetics. Meanwhile, the physical platform also tightly restricts immune receptors diffusion, which is distinct to that in solution, thereby drastically affecting immune receptor-ligand binding affinity (66). Moreover, the immune receptors also experience mechanical force induced by membrane tension and cytoskeleton contractions (23–25). The mechanical force has been reported to induce conformational changes of immune receptor and ligand to regulate their binding strength and immune functions. It is very likely that these mechanically regulated protein conformational changes could propagate across the PM and transduce toward the inside of the cell to allosterically regulate the conformation of the receptors’ cytoplasmic tails and potentially their associated kinases or other adaptor molecules. Such propagation could provide a rapid physical activation of receptor signal transduction, other than traditionally accepted biochemical ways. This mechanical regulation might be universal in immune receptors. During cross-membrane mechanical propagation, cholesterol could inevitably be integrated with force to collectively regulate the conformation of receptor’s TMD. For example, cholesterol can regulate PM tension, which in turn tunes cholesterol distribution, membrane stiffness and bending (67–69), thereby inducing immune receptors ectodomain conformational changes and TMD titling. Cholesterol could potentially prevent or facilitate the TMD tilting, which may be dependent on how cholesterol dynamically interacts with receptor’s TMD. Collectively, the mechano-biochemical coupling could contribute to the conformational changes, triggering and clustering of immune receptors.

The PM provides a physical platform for TCR/CD3 complex to sense antigens (66). On this platform, TCR inevitably experiences external mechanical forces when T cells contact the antigen-presenting cell (APC) or migrate on the APC and the extracellular matrix (ECM), and the internal mechanical force produced by dynamic cytoskeleton contraction during T-cell searching for foreign antigens on the APC or membrane bending tension upon T-cell/APC contact formation (23, 70–73). For TCR recognition of pMHCs, the mechanical force can prolong the bond lifetimes for agonistic antigens but not antagonists by selectively inducing conformational changes (**Figure 1C**) of the agonistic pMHC to initiate the formation of new hydrogen bonds (electrostatic attraction between the hydrogen atom and negatively charged nitrogen or oxygen atom) (72, 73). This force induced by pMHC/TCR binding inevitably increases local membrane tension and further induces membrane bending, which might disrupt the interactions between CD3 polybasic regions and negatively charged lipids of the inner leaflet to expose ITAM motifs for Lck to phosphorylate and further trigger downstream signaling.

PM stiffness can also regulate the antigen discrimination of BCRs. BCR has stringent affinity discrimination when contacting with rigid APC PM during the invagination of antigens (74). Also, PM shape can regulate BCR stimulation by affecting the formation of BCR microclusters (75). On the PM platform, the mechanical force provides multiple effects on different isotyped BCRs, which influences the activation sensitivity of BCR by pathological antigens (antigens that can induce a specific immune response to cause the infectious, allergic or autoimmune diseases) (76). Low mechanical force (<12 pN) is enough to trigger the activation (e.g. BCR, pSyk, pPLCγ2, and pTyr clusters) of IgG- or IgE-BCR on memory B cells, but not IgM-BCR on mature naive B cells (76).

Besides, PM curvature causes the redistribution of FcϵRI (77, 78). FcϵRI bond with IgE always locates at the contact membrane regions which are less curved (79). Similarly, FcγRIIA also can be regulated by the mechanical force under the physiological flow conditions, which facilitates the capture of neutrophils directly by endothelial cells (80). Moreover, the recognition of FcγRII and FcγRIII to IgG is influenced by the anchor patterns (e.g. GPI and transmembrane domain) on the immune cell PM (81, 82).

It has been reported that high membrane tension or mechanical force helps CD28 (another costimulatory receptor of TCR complex) to facilitate TCR signaling on the PM platform (83, 84). Also, LFA-1/ICAM-1 interaction is affected by the mechanical force to regulate T cell migration (85–88). For natural killer cells, PM stiffness can regulate the NKG2D/MICA interaction to determine the cell cytotoxic activity (89).

## Electrical Potential Regulation and Its Coupling With Mechanical and Biochemical Cues

The PM electrical potential, which is commonly overlooked in the immunology field, is another essential biophysical factor that also potentially regulates immune receptor functions. It is generally defined as the electric potential difference between the intracellular and extracellular solution. PM potential depolarization can facilitate the opening of Ca^2+^ channels and initiate the mitotic activity to regulate the activation and proliferation of lymphocyte cells (90–92). The regulation of PM potential on neural activity and the networks has been widely reported. Its molecular regulation mechanism mainly divides into three ways (93). First, TMD conformational change of the voltage-dependent ion channels is triggered by PM potential depolarization (94). Considering that the TCR complex TMD contains several charged residues buried in the lipid bilayer, we propose that PM potential depolarization, which is triggered by T cell activation (95), might tilt the conformation of TCR TMD to further allosterically regulate the dissociation of CD3 tails from the inner leaflet of PM and activate intracellular downstream signaling (**Figure 1D**). However, this depolarization-induced TCR allosteric activation needs to be further investigated with detailed biophysical investigation. Second, ion influx, an indirect effect of depolarization, regulates transmembrane proteins. The possible mechanism might be that ion influx regulates ligand binding and tyrosine phosphorylation (96, 97). It has been reported that Ca^2+^ influx disrupts the interactions between CD3 cytoplasmic polybasic regions and negatively charged lipids to favor CD3 ITAMs phosphorylation. However, whether PM potential might directly tune CD3 ITAMs phosphorylation is still unknown. Third, the electro-osmosis or electrophoresis induced by local electric fields, re-distributes transmembrane protein on the PM (98). Like neural synapses, the immune synapses might also exist this electromigration to regulate immune receptor distribution pattern, which favors the recognition of APCs by T cells.

Notably, PM surface usually exists a ~2 nm electrical double layer (EDL, a layer formed by freely diffusing electrolyte ions in the nanometer range of the charged surface), which is regulated by lipid distribution and intracellular/extracellular ions concentration (16). Key proximal regions of TMD, usually containing acidic or basic amino acids and locating in the EDL, might respond to the electrical potential change to regulate immune receptor conformations. For example, Ca^2+^ influx of T cell activation indeed changes the ion distribution and reduces the interaction between the cytoplasmic domain of CD3 or CD28 and EDL, activating downstream signal transduction.

PM potential can also affect other chemical and biophysical properties, such as lipid distribution, membrane fluidity, tension, and curvature of PM (99, 100), dynamically changing the mechanical and biochemical environment where immune receptors reside (23). For example, PM potential depolarization can induce changes in PM curvature and tension, which could further regulate the mechano-dependent behavior of immune receptors, such as the conformational changes and ligand binding kinetics (23, 100, 101). Meanwhile, the depolarization also reduces the lateral diffusion of membrane components (e.g. cholesterol, phospholipids, and protein) to affect the formation of lipid rafts, receptor microclusters, and microvilli (12, 99, 102, 103), all of which are crucial for immune receptor activation (12, 26, 28).

Inversely, the compositions of lipids and cholesterols can directly affect the charge distribution on the PM surface, which further determines the PM potential (12, 16, 30). PM tension and curvature might also tune immune cell PM potential through mechanosensitive Piezo1 channel (104), causing a series of related regulation on immune receptor activation. However, the detailed mechanism still needs further investigation.

Based on the above elaboration, the PM platform provides mechanical-electric-chemical coupling to synergistically regulate immune receptor-ligand recognition, conformational changes, and cross-membrane activation. This is also exciting to be investigated in the future.

## Conclusion

In recent decades, the regulation of immune cell PM chemical properties on the receptor activation has been broadly investigated, and some of them have been revealed. However, many other PM biophysical effects (e.g. membrane tension and electrical potential) have not been clearly examined. Especially, whether and how biophysical-chemical cues couple together to tune receptors, and their molecular mechanism of these regulation patterns all need to be further investigated. Answering all these above questions will improve our understanding of immune receptor activation, especially TCR, thus contributing to immunotherapies development [e.g. chimeric antigen receptor (CAR) T-cell design].

## Author Contributions

WC conceived the writing. TZ wrote the first version of the manuscript and prepared the figures. WH and WC revised the paper. All authors contributed to the article and approved the submitted version.

## Funding

WC is funded by grants from the Ministry of Science and Technology of China (No. 2017ZX10203205), and the National Natural Science Foundation of China (No. 31971237). WH is funded by grants from The National Natural Science Foundation of China (No. 12002307) and China Postdoctoral Science Foundation (No. 2020M671697).

## Conflict of Interest

The authors declare that the research was conducted in the absence of any commercial or financial relationships that could be construed as a potential conflict of interest.
